# Fontes para uma história da energia nuclear no Brasil: o arquivo de Witold Lepecki

**DOI:** 10.1590/S0104-59702026000100035

**Published:** 2026-10-05

**Authors:** Bernardo Soares Pereira

**Affiliations:** i Professor, Instituto Federal de São Paulo. Hortolândia – SP – Brasil. bernardo.soares@ifsp.edu.br

**Keywords:** Energia nuclear, Witold Lepecki (1936-2023, História das ciências, Tecnologia nuclear, Nuclear energy, Witold Lepecki (1936-2023, History of sciences, Nuclear technology

## Abstract

O artigo tem como objetivo apresentar o arquivo pessoal de Witold Lepecki, sob a guarda do Museu de Astronomia e Ciências Afins, no Rio de Janeiro. São apresentados brevemente aspectos da trajetória de Lepecki e sua contribuição para o desenvolvimento da ciência nuclear e para a execução do programa nuclear brasileiro, além de um sucinto histórico do arquivo. A documentação é apresentada a partir dos grandes eixos temáticos identificados durante um levantamento preliminar. Destaca-se que este trabalho constitui uma aproximação inicial ao acervo, com o intuito de divulgar sua riqueza documental e incentivar novas pesquisas, dada sua importância para a história das ciências e das técnicas no Brasil.

## Abstract (en)

This article aims to present Witold Lepecki’s private archive, currently housed by the Museu de Astronomia e Ciências Afins, in Rio de Janeiro. The text briefly presents Lepecki’s trajectory and his contribution to the development of nuclear science and the implementation of the Brazilian nuclear program, in addition to a concise history of the archive. The documentation is divided into three large thematic axes identified during a preliminary survey. It is worth noting that this study constitutes an initial approach to the collection, with the purpose of publicizing its documental richness and stimulating new studies, given its importance to the history of sciences and techniques in Brazil.

O artigo tem como objeto de análise o arquivo de Witold Lepecki, que está sob a guarda do Museu de Astronomia e Ciências Afins (Mast). Sem a pretensão de realizar um inventário do acervo – que, até o momento, permanece desorganizado e ainda não foi alvo de interesse de pesquisa –, o trabalho tem o duplo objetivo de apresentar, em linhas gerais, os principais temas presentes na documentação do arquivo e de indicar possíveis lacunas historiográficas que ele poderia ajudar a preencher, considerando seu valor para os estudos sobre a política nuclear brasileira.

A relevância dos arquivos pessoais de cientistas para a produção do conhecimento histórico acerca das ciências e das técnicas já tem sido ressaltada por diversos autores. Além de se interessar pela documentação produzida pelas instituições responsáveis pela produção e pelo financiamento da pesquisa científica, a história das ciências, conforme nos lembra Juliana Silva (2022), também atenta para a produção documental dos sujeitos atuantes no desenvolvimento das atividades científicas, ou seja, os cientistas.

Como aponta Maria Celina Soares de Mello e Silva (2007), contudo, os limites entre o pessoal e o institucional nos arquivos dos cientistas nem sempre são fáceis de serem estabelecidos. Essa ambiguidade é evidente no caso do arquivo de Witold Lepecki, um cientista que ao longo de sua carreira assumiu diversas posições de chefia nas instituições em que atuou. Embora classificado como um fundo pessoal, entre seus documentos coexistem aqueles de natureza administrativa ou institucional e aqueles de preponderante natureza técnica, como apresentações de seminário, artigos e relatórios. Por outro lado, estão ausentes registros geralmente associados aos arquivos pessoais tradicionais, como correspondência íntima, fotografias familiares ou registros escolares. Por essa característica do arquivo, talvez seja mais prudente referirmo-nos a ele não como um “arquivo científico”, expressão considerada inadequada por Ana Maria de Almeida Camargo (2006, p.13), mas – como a mesma autora sugere – um “arquivo de interesse para a ciência ou para a história da ciência”.

Nas 215 caixas^1^ do Arquivo Witold Lepecki, encontram-se documentos que cobrem temas centrais da política nuclear brasileira, como o Acordo Brasil-Alemanha Ocidental de 1975, as pesquisas nacionais de desenvolvimento de reatores, as negociações internacionais para a criação de uma indústria nuclear no Brasil e os debates sobre segurança de reatores no contexto após o desastre de Chernobyl.

Além dessa introdução, este artigo conta com duas seções e as considerações finais. A primeira apresenta elementos da trajetória profissional de Lepecki e um breve histórico da constituição de seu arquivo. As informações utilizadas foram extraídas de seu currículo profissional enviado à equipe do Mast na ocasião da doação do acervo e enriquecidas com seu depoimento ao projeto “Memória histórica e estratégica da energia nuclear”, desenvolvido pelo Centro de Pesquisa e Documentação de História Contemporânea do Brasil da Fundação Getúlio Vargas.

Acerca desse tópico, convém destacarmos duas limitações. Em primeiro lugar, trata-se apenas de uma apresentação geral da vida profissional de Lepecki, não de uma construção biográfica. Em segundo, essas informações se limitam ao período anterior à doação do arquivo. No que se refere ao histórico da constituição do arquivo, foi elaborado com base na correspondência via *e-mail* trocada por Lepecki e a equipe do Mast, entre o final de 2009 e o início de 2010, quando ocorreu a doação. Considerando que Lepecki faleceu em 2023, é natural que esta seção apresente lacunas sobre suas duas últimas décadas de vida.

Na terceira parte do artigo é apresentado um panorama geral do acervo e são sugeridas algumas possibilidades de pesquisa. Não se pretende aqui realizar um inventário ou antecipar a descrição arquivística formal do fundo. As considerações que serão feitas resultam do olhar de um historiador que investiga a história da energia nuclear e reconhece nesse arquivo uma fonte rica e inexplorada. Da mesma forma, as sugestões de temas de pesquisa refletem as lacunas historiográficas percebidas pelo autor.

Em que pesem essas limitações, as reflexões reunidas podem contribuir para futuros trabalhos de organização e tratamento arquivístico. Além disso, espera-se que a apresentação e discussão do acervo possa contribuir para o avanço dos mais variados campos das ciências humanas que se interessam por temas relacionados à história da ciência e da tecnologia no Brasil, especialmente ao campo da ciência nuclear.

## Witold Lepecki: trajetória profissional e constituição de seu arquivo

Witold Piotr Stefan Lepecki nasceu em 2 de maio de 1936, em Varsóvia, Polônia, mudando-se com sua família para o Brasil no ano de 1939, no contexto das invasões alemãs e soviéticas que antecederam ao início da Segunda Guerra Mundial. Inicialmente instalados em Curitiba, os Lepecki transferiram-se para Belo Horizonte em 1944, onde Witold fez seus estudos e iniciou sua vida profissional (Lepecki, 2010).

Após realizar os estudos secundários no Instituto Padre Machado, Lepecki cursou engenharia civil na Universidade de Minas Gerais entre os anos de 1954 e 1957. Entre os motivos de ter optado pela carreira, Lepecki destacaria as possibilidades profissionais e financeiras que ela poderia proporcionar, superiores à formação em matemática ou física, campos de interesse nos quais continuaria realizando estudos paralelos (Lepecki, 2010).

No último ano da faculdade foi estagiário da Companhia Energética de Minas Gerais, onde foi efetivado em janeiro de 1959, mas só permaneceu até maio daquele ano. É desse período a orientação de Lepecki para as ciências nucleares, tendo ingressado em março no curso de especialização sobre ciências e técnicas nucleares oferecido pela Comissão Nacional de Energia Nuclear (Cnen), concluído em dezembro de 1960. Posteriormente, o título foi reconhecido como equivalente ao de mestre em engenharia nuclear.

Em 1961 iniciou seu trabalho como engenheiro no Instituto de Pesquisas Radioativas (IPR), da Universidade de Minas Gerais. No IPR trabalhou em diversos grupos de relevância para as ciências nucleares no Brasil, como o que desenvolveu pesquisas no reator Triga e, posteriormente, na Divisão de Engenharia de Reatores, dentro da qual se destacou o chamado Grupo do Tório.

Acerca do último grupo, destaca-se seu caráter pioneiro no desenvolvimento dessa linha de reatores. Por não ser um material físsil, o tório exige ser misturado com o urânio-235 ou o plutônio-239. Pelo fato de este último ser um subproduto dos reatores da linha de urânio natural e água pesada, o Grupo do Tório defendia que o país primeiro ingressasse em um projeto de desenvolvimento de reatores de urânio natural para, posteriormente, ter condições de desenvolver sua própria linha. Contudo, o fato de a alta esfera governamental, com apoio da Cnen, ter optado pela linha de urânio enriquecido, acabou desestimulando as pesquisas de reatores a tório, atitude criticada por setores da comunidade científica nacional. Como consequência, o Grupo do Tório foi descontinuado em 1972.

Entre 1964 e 1965, Lepecki foi enviado à França pela Cnen para se especializar na área de otimização de reatores e física de reatores. Naquele país, realizou em paralelo o seu doutorado, tendo obtido o título de *docteur 3ème cycle* em 1965, com uma tese sobre o estudo de reatores a grafite e plutônio.

Retornando ao Brasil, desempenhou atividades de chefia no IPR, liderando a Divisão de Engenharia de Reatores, que nesse momento se expandiu de dez para cinquenta engenheiros especializados e sob o qual atuava o Grupo do Tório. Paralelo às atividades do IPR, Lepecki ainda auxiliou o chamado Grupo Lane, formado por meio da associação da Agência Internacional de Energia Nuclear, da Cnen e da Eletrobrás, chefiado pelo engenheiro norte-americano James Lane, que foi responsável por redigir um documento que balizaria a tomada de decisão da construção da usina de Angra 1.

Com o fim do Grupo do Tório, foi designado para se incorporar à recém-criada Companhia Brasileira de Tecnologia Nuclear (CBTN),^2^ onde assessorou Carlos Syllus, então diretor de Tecnologia e Desenvolvimento, entre 1972 e 1975. Desse período, destaca-se sua participação nas atividades de planejamento dos três institutos de pesquisas vinculados à CBTN (Instituto de Pesquisas Radioativas, Instituto de Engenharia Nuclear e Instituto de Radioproteção e Dosimetria), a coordenação executiva do estudo sobre a capacidade da indústria brasileira de fabricar componentes para centrais nucleares, elaborado pela CBTN em parceria com a empresa americana Bechtel, em 1973, e a participação nas negociações do acordo nuclear entre Brasil e Alemanha Ocidental, em 1975.^3^

A respeito do acordo com a Alemanha, Lepecki esteve diretamente envolvido tanto nas negociações referentes à fábrica de componentes pesados, que posteriormente viria a se concretizar com a criação da Nuclebrás Equipamentos Pesados (Nuclep),^4^ quanto nas negociações sobre o processo de enriquecimento de urânio, nesse último ponto tendo ocupado a direção da Nustep,^5^ empresa sediada na Alemanha.

Em 1979, ao retornar da Alemanha, foi convidado pelo então presidente da Nuclebrás, Paulo Nogueira Batista, para trabalhar na área técnica da Nuclebrás Engenharia S.A. (Nuclen),^6^ onde se ocupou da assimilação dos projetos dos sistemas nucleares com o objetivo de fixar no Brasil a tecnologia alemã. No interior dessa empresa ocuparia diversos cargos de chefia ao longo das décadas de 1980 e 1990, até a reorganização do setor nuclear e a criação da Eletronuclear, empresa na qual chefiou o projeto “Determinação do *know-how* tecnológico”.

Nos anos 1980, após o acidente de Three Mile Island e o aumento da preocupação com a questão de segurança em usinas nucleares, agravada com o acidente de Chernobyl, Lepecki foi convidado pelo presidente da Agência Internacional de Energia Atômica (AIEA) para participar do International Nuclear Safety Group (Insag). Desse período, datam diversas conferências e publicações do autor acerca do tema de segurança de reatores, que o acompanhou até pelo menos o início dos anos 2000, últimos anos registrados em seu arquivo.

Acerca do arquivo, as tratativas de sua doação ao Mast se iniciaram no último mês do ano de 2009. Os primeiros contatos entre Lepecki e a instituição se deram entre 2005 e 2006, quando a pesquisadora Ana Maria Ribeiro de Andrade abordou diversos engenheiros no sentido de informar o interesse do museu em organizar e preservar arquivos de ciência e tecnologia. Passados alguns anos desse diálogo inicial, a pesquisadora foi procurada por Witold Lepecki, que manifestou seu interesse em doar sua documentação, que se encontrava em local pouco protegido, nas instalações da Eletronuclear, em Angra dos Reis.

Os documentos chegaram ao Mast no dia 29 de janeiro de 2010, agrupados em 35 caixas de grandes dimensões. Ressalta-se que a equipe do museu não tinha maiores informações acerca do acervo, contando apenas com indicações mais genéricas sobre a existência de documentos do Grupo do Tório. Ao chegar ao Mast, os documentos passaram por um processo de higienização, sendo posteriormente agrupados em 215 caixas padrão da instituição, respeitando a ordem das caixas enviadas por Lepecki.

## Panorama geral do acervo e possibilidades de pesquisa

A maior dificuldade de pesquisa no arquivo de Witold Lepecki consiste no fato de ele não estar organizado. Na ocasião de sua chegada ao Mast houve uma tentativa de organização, mas que não foi levada adiante. Além disso, não foi fornecido pelo próprio Lepecki qualquer documento informando a natureza do material que estava sendo doado. Embora em algumas poucas pastas haja indicações, para a grande maioria do acervo não há informação adicional. Por outro lado, ressalta-se que a falta de organização é amenizada pela boa conservação do acervo e pela presteza da equipe do Mast em auxiliar os pesquisadores.

Dessa forma, o pesquisador que se interessar pelo arquivo terá que abrir todas as caixas do acervo, que preenche praticamente toda uma estante da reserva técnica do museu, no intuito de aferir se há alguma documentação que o interesse. Até se iniciar essa pesquisa, ninguém havia se aventurado à tarefa.

**Figura 1:**
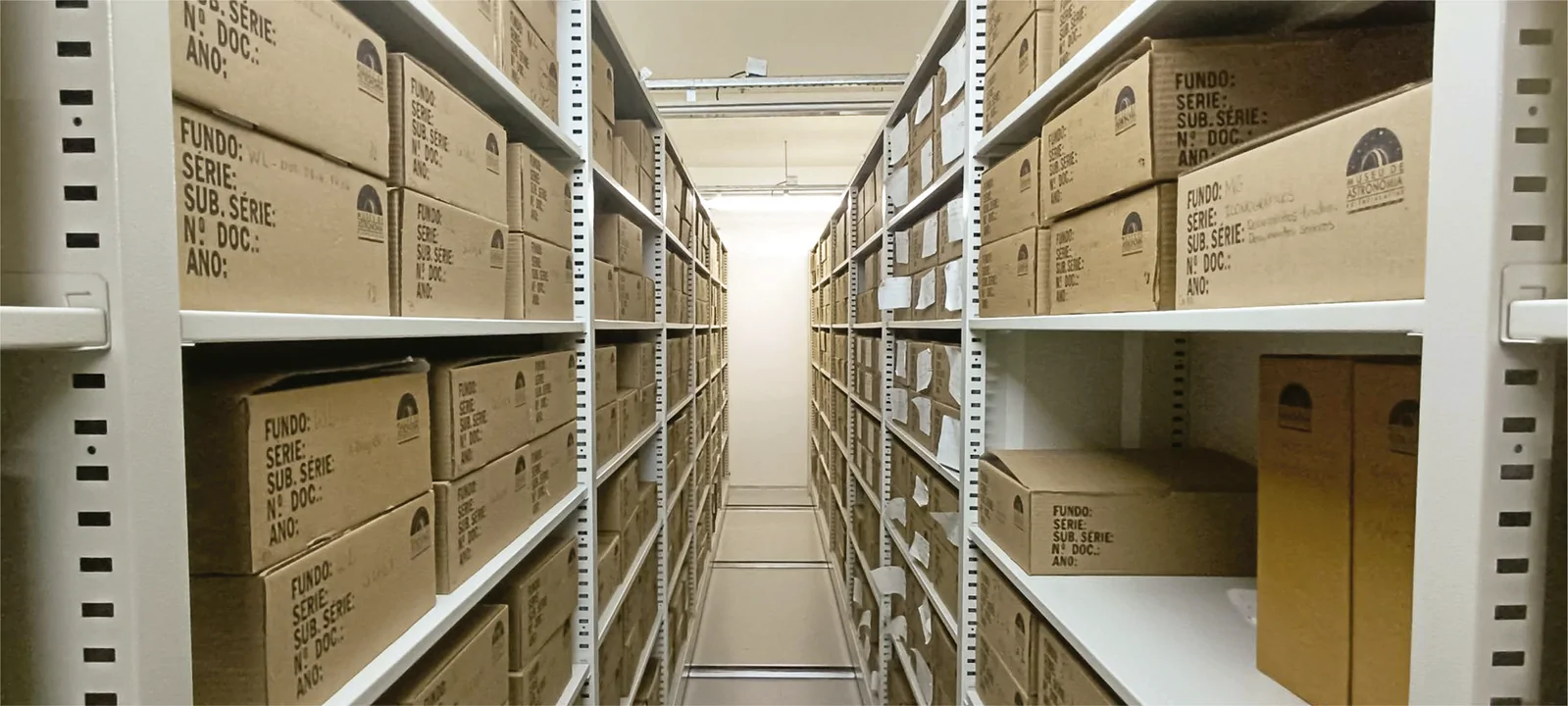
Arquivo do Mast. O acervo de Witold Lepecki está armazenado na estante à esquerda (Fonte: foto do autor)

Durante a produção do trabalho que resultou na confecção deste texto, foram feitos dois períodos de visitas ao Mast, em 2024 e 2025. Na medida em que as caixas foram abertas, registraram-se áudios que posteriormente foram sistematizados em planilha. As considerações eram breves, limitando-se a observações sobre o conteúdo geral de cada caixa, principal tema que os documentos abordavam e data aproximada de produção.

Destaca-se que o acervo é composto majoritariamente por documentos em suporte papel, com exceção de alguns materiais em suporte magnético, como um conjunto de disquetes na caixa 92, e de alguns documentos iconográficos, entre os quais se destacam as fotografias que se encontram em um fichário na caixa 108. Nesse conjunto, é possível ver registros do transporte dos componentes pesados das usinas de Angra 2 e 3, desde sua saída da Alemanha até sua chegada ao estado do Rio de Janeiro.

**Figura 2:**
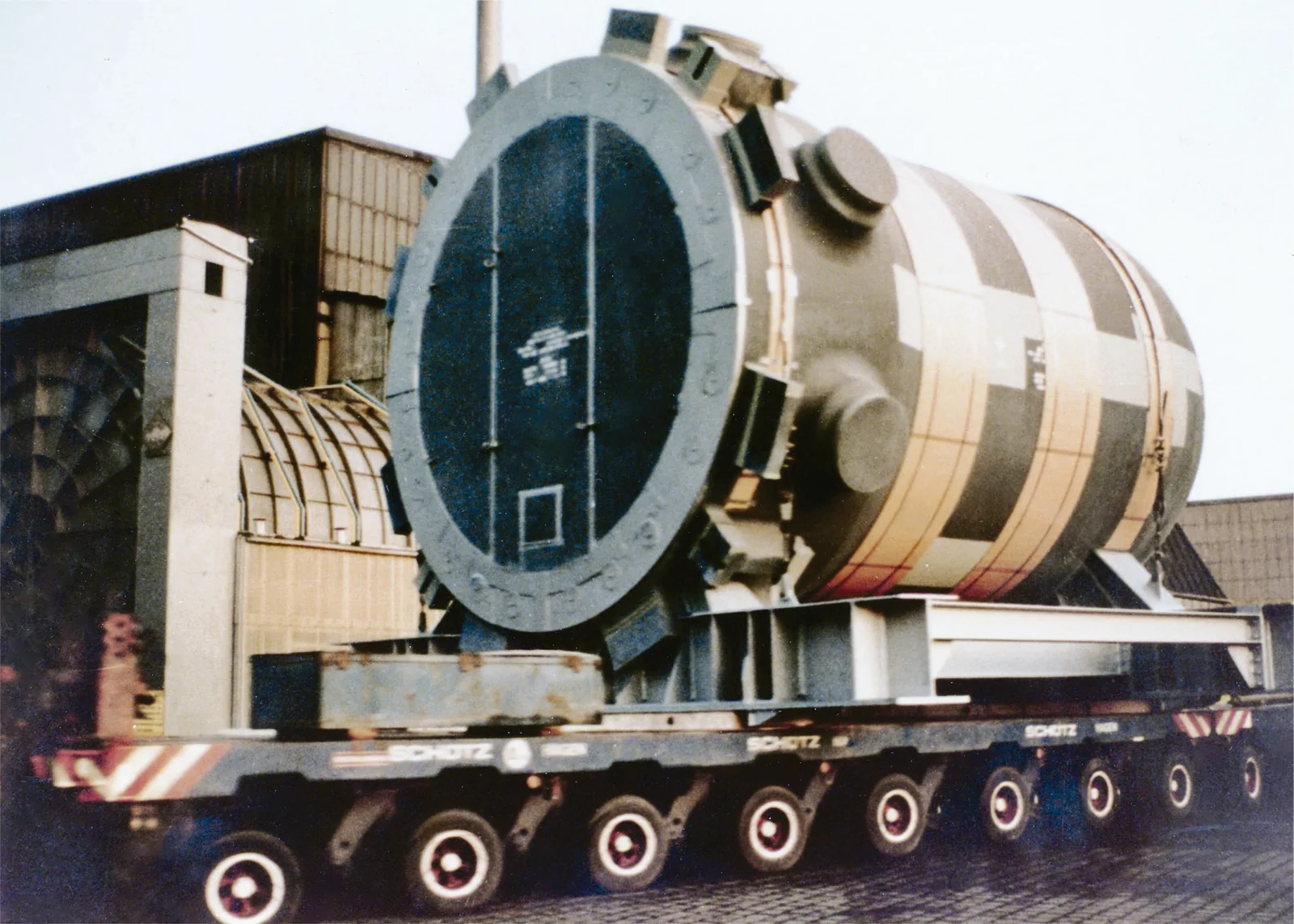
Vaso do reator de pressão de Angra 2 (Fonte: Arquivo Witold Lepecki, caixa 108)

Sobre a tipologia documental, registra-se sua ampla diversidade. São abundantes os relatórios técnicos, especialmente do período em que Lepecki atuou no IPR, bem como sua participação nas empresas vinculadas à Nuclebrás. Também se destacam os documentos relativos à segurança de reatores, incluindo transparências de apresentações em eventos científicos, documentos técnicos da AIEA e do Insag.

Conforme comentado, a documentação institucional é a marca do acervo. Predominam documentos como atas de reuniões, ofícios, memorandos e aqueles relativos à cooperação internacional, como registros de missões de pesquisadores brasileiros no exterior. Essa constatação torna o acervo rico para aqueles que desejam se aprofundar na história das instituições brasileiras ligadas ao setor nuclear, como o IPR, a Cnen e a Nuclebrás.

Sobre a tentativa de estabelecer um levantamento temático preliminar da documentação, destaca-se que não era incomum observar documentos de temáticas diferentes e produzidos em um grande intervalo de tempo dentro de uma mesma caixa. Dessa maneira, uma tentativa de apresentação mais geral e em conjunto das caixas, como a que se faz aqui, incorre em incontornáveis imprecisões, sendo superada apenas pelo rigoroso trabalho de realização de inventário por uma equipe de arquivistas e historiadores.

Sem desconsiderar essas limitações, os grandes blocos temáticos e sua representatividade no interior do arquivo podem ser vistos no Gráfico 1.

**Figura 3:**
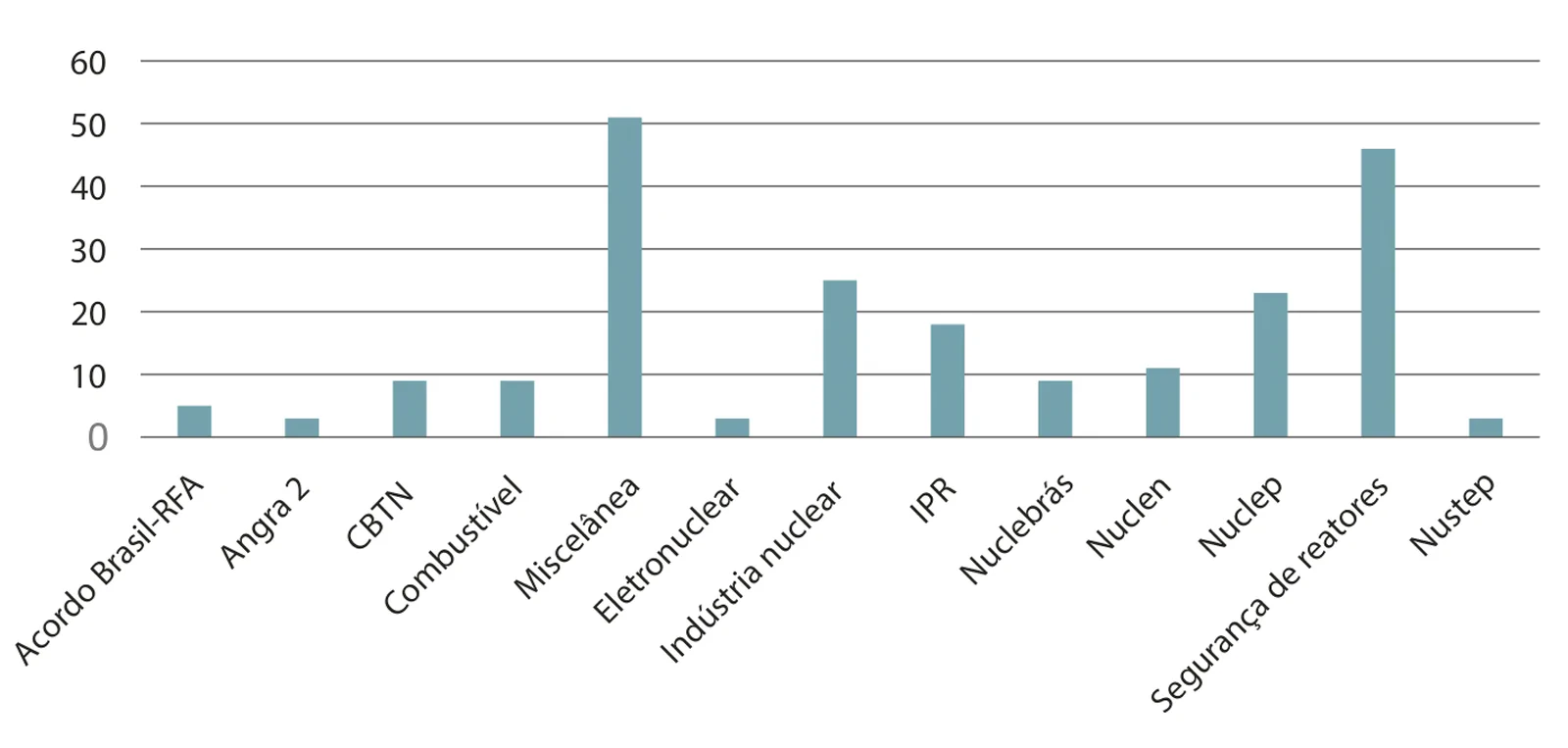
Gráfico com blocos temáticos predominantes nas caixas do Arquivo Witold Lepecki (Fonte: elaboração do autor)

Sobre os dados apresentados no gráfico, algumas considerações são importantes. Primeiramente, a categoria “Miscelânea” reúne uma documentação múltipla, de classificação incerta em uma primeira aproximação e com pouca conexão entre si. Foram incluídas nessa categoria caixas com encartes institucionais de empresas do setor elétrico e nuclear de diversos países, disquetes, dezenas de impressões de artigos de opinião publicados na internet, recortes de jornais, impressões de *e-mails*, um guia de usuário do Corel Draw, agendas pessoais de Lepecki, apresentações em seminário, bem como artigos de outros pesquisadores. Essa diversidade revela a complexidade da tipologia documental do arquivo e antecipa o desafio de uma equipe arquivista.

A segunda categoria com maior número de caixas é aquela que chamamos de “Segurança de reatores”, questão que ganhou fôlego na vida profissional de Lepecki a partir dos anos 1980. Entre esses documentos, destacam-se muitos estudos sobre o caso de Chernobyl, artigos científicos, transparências de apresentações em eventos internacionais, relatórios da AIEA e documentos relacionados à atuação de Lepecki no Insag.

A terceira categoria em destaque refere-se a “Indústria nuclear”. Acerca desse assunto, podem ser encontrados documentos sobre a participação de Lepecki nos estudos da CBTN e da Bechtel sobre a capacidade da indústria brasileira de produzir equipamentos para centrais nucleares, bem como diversos relatórios sobre as fábricas brasileiras e registros da missão técnica alemã que em 1973 visitou diversas instalações industriais no Brasil.

Esse conjunto do acervo é particularmente rico ao abordar as negociações com fornecedores internacionais, como a Westinghouse, que, às vésperas da assinatura do Acordo Brasil-Alemanha, tentou oferecer ao governo uma tecnologia que esbarrava em restrições da política de não proliferação dos EUA (CBTN/DTD, 23 out. 1974). Há ainda documentos que permitem acompanhar a manifestação de interesse da Mitsubishi em fornecer componentes nucleares para o Brasil, como foi tentado em 1975 e que não prosperou (Nuclebrás, 21 out. 1975).

Ainda sobre a questão industrial, existem registros de reuniões com representantes da indústria nacional, como Bardella, Cobrasma, Confab e Villares. Ressalta-se que as três primeiras assinaram um acordo que lhes dava preferência no fornecimento de equipamentos pesados a serem produzidos no Brasil para as quatro primeiras centrais nucleares oriundas do acordo de 1975. Contudo, a maior parte dos componentes pesados seria fornecida pelos alemães, uma vez que o acordo previa uma nacionalização de 30% desses itens nas duas primeiras usinas até alcançar um índice de 70% nas usinas sete e oito. Por outro lado, a nacionalização dos turbogeradores, componentes de grande sofisticação tecnológica, não ultrapassaria o índice de 30%. Esses baixos índices de nacionalização foram objetos de críticas de setores da sociedade brasileira ao acordo, sobretudo quando ele apresentou seus primeiros sinais de problemas de execução, entre finais dos anos 1970 e início dos anos 1980.

Sobre essa relação com os interesses industriais alemães, é interessante ressaltar um documento da caixa 84 em que os brasileiros apresentam aos alemães o desejo de uma nacionalização mais rápida da produção dos componentes do conjunto de turbogeradores no Brasil, e os alemães expressam o desejo de fabricar turbinas hidráulicas na Nuclep, o que não estava previsto no acordo e se chocava com interesses de grupos empresariais já estabelecidos no Brasil.

Embora tenha se optado por uma categorização das caixas considerando os aspectos institucionais aos quais os documentos se referiam, pode-se afirmar que entre as categorias “Acordo Brasil-RFA”, “Nuclebrás”, “Nuclen”, “Nuclep” e “Nustep” há uma estreita relação, uma vez que todas dizem respeito ao acordo de 1975 e a seus desdobramentos.

Considerando essa configuração temática do fundo e à luz da literatura sobre a questão nuclear no Brasil, ressaltam-se dois veios de pesquisa com os quais o acervo poderia contribuir para o enriquecimento historiográfico.

### As negociações do Acordo Nuclear Brasil-República Federal da Alemanha

A literatura sobre o Acordo Nuclear entre Brasil e Alemanha Ocidental é extensa, predominando uma interpretação crítica acerca de seus principais pontos. Entre as críticas que são apresentadas, muitas feitas nos anos subsequentes à sua assinatura, estão a venda, por parte dos alemães, de uma tecnologia de enriquecimento de urânio não comprovada em escala industrial, o *jet-nozzle*, e a existência de cláusulas no acordo de acionistas das *joint ventures* que permitiam aos alemães controlarem as decisões dessas empresas mesmo detendo participação minoritária do capital investido (Brandão, 2008).

Justamente esses dois aspectos podem ser aprofundados por pesquisadores que se interessem em mergulhar na documentação do arquivo de Witold Lepecki. Acerca da constituição das *joint ventures*, Lepecki esteve profundamente envolvido nas negociações da Nuclep e da Nustep.

No caso da Nuclep, é possível observar os primeiros relatórios da missão alemã que realizou um levantamento sobre a capacidade da indústria brasileira de produzir equipamentos pesados, bem como estudos técnicos sobre os melhores sítios para a construção da fábrica da Nuclep.^7^ Contudo, o que mais pode contribuir para a ampliação do conhecimento acadêmico é a extensa documentação acerca do contrato de acionistas da empresa. Nas diversas caixas que contêm documentos sobre o tema, pode-se encontrar não somente o acordo de acionistas da empresa – que, por muito tempo, a Nuclebrás se recusou a fornecer sob alegação de sigilo industrial –, mas também a documentação das discussões preliminares que lhe deram origem.

O arquivo de Lepecki registra documentos anteriores ao acordo de Bonn, de junho de 1975, bem como os materiais relativos às quatro rodadas de negociação sobre a Nuclep ocorridas após a assinatura do acordo: (1) setembro a outubro de 1975; (2) novembro a dezembro de 1975; (3) fevereiro a março de 1976; (4) junho de 1976.

Dentre os documentos relativos às reuniões preliminares, destaca-se um conjunto de documentos datados de fevereiro de 1975, no qual já se delineavam as diretrizes da futura organização Nuclep – que, até maio daquele ano, seria denominada Componentes Pesados Nucleares Brasileiros. Já se previa, por exemplo, a criação de um comitê técnico composto exclusivamente por alemães, responsável pela decisão final quanto à qualidade dos produtos (Nuclebrás, abr. 1975).

Sobre as rodadas de negociação após a assinatura do Acordo de Bonn, é possível ver um curioso documento de recomendação à delegação brasileira que participaria da primeira rodada, no qual se estabelecia tanto os limites que os brasileiros tinham para negociar, quanto se recomendava parcimônia ao comer e beber para se manterem em boas condições para o debate.

**Figura 4:**
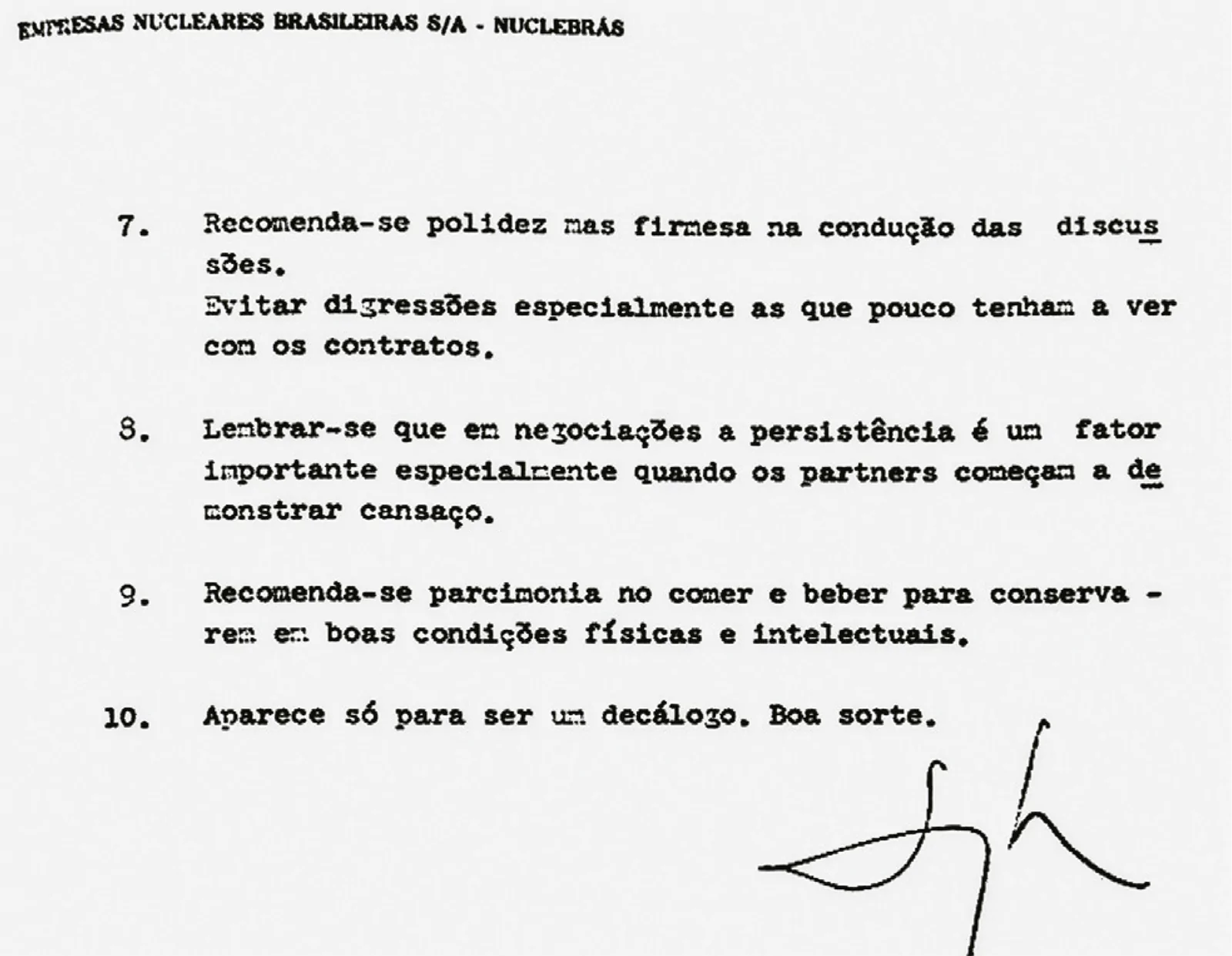
Recomendações à delegação brasileira à primeira rodada de negociações da Nuclep (Fonte: Arquivo Witold Lepecki, caixa 68, Recomendações ao Grupo, Documento da Nuclebrás)

Ainda sobre essa reunião, é possível consultar dois relatórios escritos por Lepecki (3 out. 1975, 12 out. 1975) , em que são expostos os pontos debatidos ao longo dos encontros, os avanços obtidos e os principais impasses entre as delegações. Acerca das demais reuniões, o acervo contém diversos rascunhos do contrato de acionistas com anotações manuscritas de Lepecki na margem, nas quais se podem ver observações sobre os mais diversos pontos.^8^ Há ainda agendas utilizadas por ele como cadernetas durante a terceira e a quarta rodadas de negociação.^9^

Quanto à Nustep, empresa sediada na Alemanha e que ficaria responsável pelo desenvolvimento do *jet-nozzle*, a documentação referente ao seu acordo de acionistas pode ser encontrada nas caixas 71 e 75. Já os documentos técnicos relacionados aos estudos sobre os processos de enriquecimento estão distribuídos nas caixas 85 e 160.

### Para uma história das instituições de pesquisas nucleares no Brasil: o caso do Grupo do Tório

A história das instituições de pesquisas nucleares no Brasil ainda carece de aprofundamento por parte da pesquisa científica, embora existam obras de referência sobre o Ipen (Gordon, 2003) e a Cnen (Andrade, Santos, 2013; Santos, 2009). Nesse sentido, o arquivo de Lepecki apresenta-se como uma fonte rica, contendo documentos que podem contribuir para a reconstrução de uma das mais relevantes experiências de pesquisas para o desenvolvimento de uma linha autônoma de reatores de potência: o Grupo do Tório, pertencente à Divisão de Engenharia de Reatores do IPR, naquela ocasião ainda vinculado à Universidade Federal de Minas Gerais e que funcionou entre 1965 e 1972.

O Grupo do Tório é frequentemente apontado por pesquisadores como um exemplo de tentativa de desenvolvimento de uma linha autônoma de pesquisa. As pesquisas históricas já realizadas sobre o Grupo do Tório são introdutórias, limitando-se a descrições panorâmicas de suas linhas de pesquisa e de sua importância para o desenvolvimento das ciências nucleares no Brasil (Andrade, Santos, 2005; Neves, Gordon, 2008). Além disso, pode-se observar a existência de tentativas de reconstrução do histórico desse grupo feitas por alguns de seus principais integrantes, como a elaborada por Ricardo Brant Pinheiro (1979).

No arquivo de Lepecki, encontram-se diversos documentos relativos ao IPR e ao Grupo do Tório, tais como relatórios mensais de pesquisas,^10^ textos mais gerais sobre o grupo em relação ao panorama da energia nuclear no Brasil,^11^ currículo de alguns de seus membros^12^ e uma ampla documentação de natureza técnica.^13^ Um documento interessante é uma carta de 21 de junho de 1972, enviada por Marcello de Vasconcellos Coelho, na época reitor da Universidade Federal de Minas Gerais, ao então ministro da Educação e Cultura, Jarbas Passarinho, ressaltando a perda que a universidade teria com o desmembramento do IPR e sua incorporação pela CBTN, sugerindo medidas que reduzissem o impacto dessa decisão (Coelho, 21 jun. 1972).

Em linhas gerais, acredita-se que a documentação relativa ao IPR e ao Grupo do Tório presente no arquivo de Lepecki pode contribuir de forma significativa para a construção de uma história das instituições nucleares no Brasil, bem como para a reconstrução da trajetória de uma linha de pesquisa nacional na área de reatores de potência. Em conjunto com documentos que podem ser consultados nos acervos da Cnen e do CDTN, o arquivo oferece aos historiadores uma fonte inédita e promissora de pesquisa.

## Considerações finais

O presente artigo teve como objetivo apresentar uma primeira aproximação do arquivo de Witold Lepecki, engenheiro nuclear com destacada participação tanto nas pesquisas científicas nesse campo quanto na execução das políticas públicas com a finalidade de implantar uma indústria nuclear no país. Sua relevante trajetória profissional perpassou por importantes instituições nacionais da área, como o IPR, a CBTN e a Nuclebrás (e suas subsidiárias), além de organismos internacionais (como a AIEA e o Insag), o que confere ao acervo uma pluralidade institucional de grande valor.

Embora o arquivo ainda careça de uma organização arquivística que permita uma consulta satisfatória aos pesquisadores, acredita-se que o levantamento preliminar realizado durante a pesquisa revelou a presença de núcleos documentais de potencial relevância acadêmica. Os documentos previamente localizados, em conjunto com outras fontes já disponíveis, podem lançar luz sobre debates clássicos na historiografia da ciência e da tecnologia no país, como a discussão referente ao acordo entre Brasil e Alemanha, e avançar em lacunas existentes, como sobre o Grupo do Tório. Ainda que alguns desses temas tenham sido brevemente adiantados neste artigo, seu aprofundamento deve ser objeto de futuros trabalhos.

Para além da divulgação do acervo, são sugeridas linhas de pesquisa para serem aprofundadas, apenas a título de exemplificação. A documentação pode ser mobilizada por distintos campos acadêmicos, como os estudos acerca da diplomacia científica, da proliferação internacional de tecnologias sensíveis no contexto da Guerra Fria, da história institucional de centros de pesquisas no Brasil e da relação entre Estado e indústria. Seguramente, outras possibilidades poderão ser adicionadas por futuros pesquisadores.

Finalmente, espera-se que o artigo possa contribuir para reforçar a importância da preservação dos arquivos pessoais e de interesse para a história da ciência. Em que pese a relevância pessoal de Witold Lepecki, seu arquivo ultrapassa o interesse biográfico, fornecendo elementos importantes para a escrita de uma história da ciência e da tecnologia brasileira.

## Data Availability

Não estão em repositório.

## NOTAS

1 Apesar de ser contabilizado como 214, o acervo possui 215 caixas, uma vez que a caixa 152 tem notação duplicada.

2 Criada em dezembro de 1971, subordinada à Cnen. Seus objetivos iam desde a realização da pesquisa de minérios nucleares ao desenvolvimento de uma indústria nuclear. Em dezembro de 1974 foi transformada na Nuclebrás.

3 Assinado em 27 de junho de 1975, o acordo previa a construção de oito usinas nucleares no Brasil e a transferência da tecnologia do ciclo completo do combustível, desde o enriquecimento ao reprocessamento do combustível irradiado. Para sua execução, era prevista a criação de diversas empresas cujo capital acionário se dividiria entre brasileiros e alemães.

4 A Nuclep foi uma das *joint ventures* criadas a partir do acordo entre Brasil e Alemanha Ocidental. Sua composição acionária era 75% da Nuclebrás e 25% de um consórcio formado por KWU, GHH e Voest Alpine.

5 Outra *joint venture* criada em decorrência do Acordo de 1975, cuja composição acionária era de 50% da Nuclebrás e 50% da Steag. Instalada na Alemanha, tinha como missão desenvolver o método do *jet-nozzle* em escala industrial.

6 Empresa também criada em decorrência do acordo com a Alemanha, sua composição acionária era 75% da Nuclebrás e 25% da KWU.

7 Ver caixas 64, 72, 83, 91, 118.

8 Ver caixas 60, 62, 68, 73 e 74.

9 Ver caixa 56.

10 Caixa 10.

11 Caixa 11.

12 Caixa 18.

13 As caixas que mais se referem ao IPR e ao Grupo do Tório são: 10, 11, 12, 14, 17, 18, 23, 40, 65, 96, 106, 149, 150, 154, 159, 196.
